# Strategies to improve the thermoelectric performance of iron silicide-based materials

**DOI:** 10.1080/14686996.2025.2585555

**Published:** 2025-11-10

**Authors:** Sopheap Sam, Sreypich Say, Kosuke Yamazaki, Hiroshi Nakatsugawa

**Affiliations:** a Department of Industrial and Mechanical Engineering, Faculty of Electrical Engineering, Institute of Technology of Cambodia, Phnom Penh, Cambodia; b Research and Innovation Center, Institute of Technology of Cambodia, Phnom Penh, Cambodia; c University of Puthisastra, Phnom Penh, Cambodia; d Graduate School of Engineering Science, Yokohama National University, Yokohama, Japan

**Keywords:** Iron silicide, structural properties, transport properties, thermoelectric materials

## Abstract

Iron silicide (β-FeSi_2_) has attracted considerable interest as a sustainable thermoelectric material due to its abundance, non-toxicity, and environmental compatibility. Their conduction flexibility allows a wide range of dopants to tune transport behavior, creating opportunities for improved performance. However, dopant solubility limits and the formation of secondary phases remain key challenges. In this article, we highlight recent advances in strategies to enhance the thermoelectric performance of β-FeSi_2_-based materials and discuss the interplay between phase evolution, electrical, and thermal transport. We also outline prospects that may unlock further improvements, offering pathways toward higher thermoelectric efficiency in this material system.

## IMPACT STATEMENT

This review covers the observation of structural transition and strategies to enhance electrical and thermoelectric properties of metal-doped iron silicides, providing insights for future research to improve material performance.

## GRAPHICAL ABSTRACT

**Figure:**
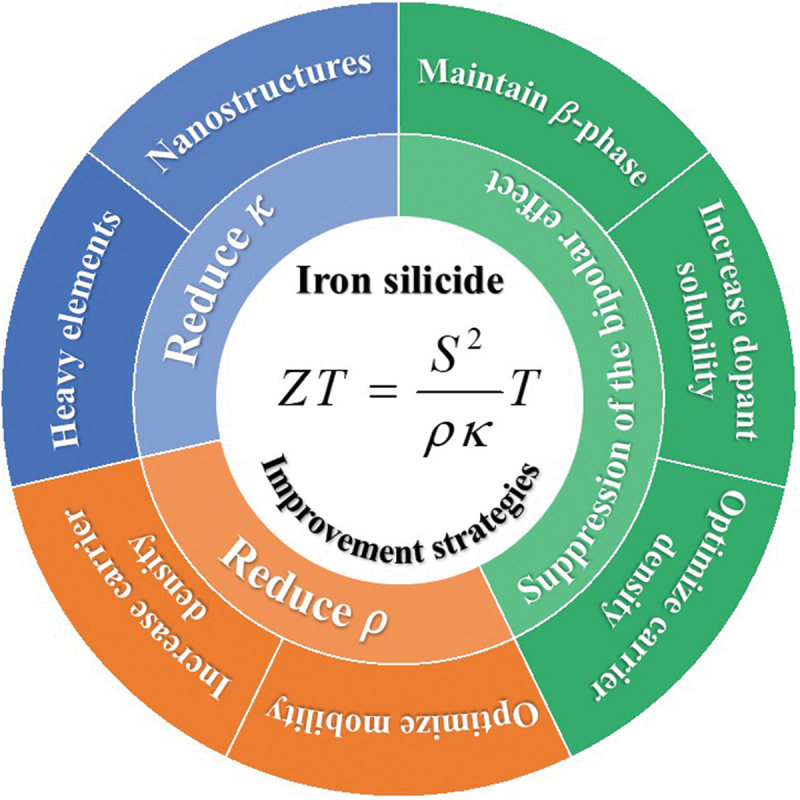

## 1. Introduction

**Figure 1.**
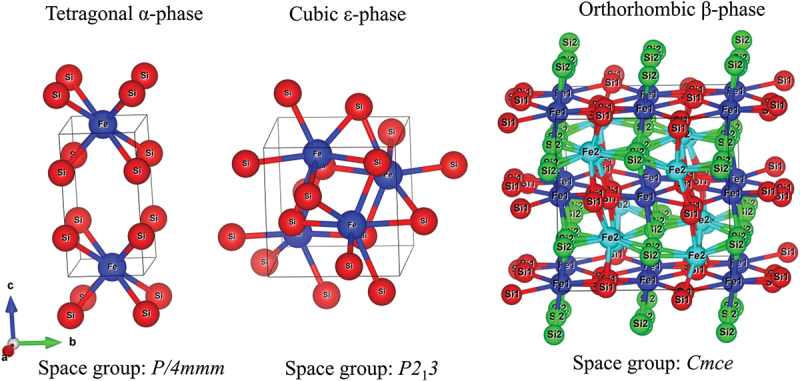
Three-dimensional crystal structures of iron silicide. The left and middle panels are metallic phases, the tetragonal α-phase and cubic ε-phase. The right panel is the structure of the semiconducting β-phase.

Thermoelectric (TE) generators are a solid-state device that could harvest waste heat and directly convert it into electricity without any moving parts or chemical pollution released to the environment [1,2]. The performance of the TE device is primarily related to the materials’ performance, as measured by the dimensionless figure of merit (*ZT*). The *ZT* value of a material is defined by: *ZT*=*S*^2^*ρ*^−1^*κ*^−1^*T*, where *S* is the Seebeck coefficient, *ρ* is the electrical resistivity, *T* is the temperature, and *κ* is the total thermal conductivity dominated by electronic and lattice thermal conductivity (*κ* = *κ*_*e*_*+ κ*_*l*_). In addition, the power factor (*PF*) of a material is expressed by: *PF* = *S*^2^*ρ*^−1^ [3]. The improvement of *ZT* value can be achieved by increasing *PF* and reducing *κ*. Typically, materials with an acceptable conversion efficiency, such as Pb, Bi, and Te in PbTe and Bi_2_Te_3_ [4–10], are either rare or toxic. Therefore, researchers attempted to optimize the transport properties of abundant and less/non-toxic compounds such as oxides [4–7], Heusler [8–13], and silicide [14–23]. Among silicide compounds, iron silicide is a promising TE material due to its oxidation resistance, thermal stability, and ability to operate at high temperatures [23–25]. As shown in Figure 1, iron silicide can be crystallized in three phases, namely tetragonal α-phase (α-Fe_2_Si_5_, *P4/mmm* space group) [26], cubic ε-phase (ε-FeSi, *P2*_1_*3* space group) [27], and orthorhombic β-phase (β-FeSi_2_, *Cmce* space group) [28–30]. Based on the Piton and Fay [15], the semiconducting β-phase can be obtained at growth temperatures below 1259 K, and its formation is influenced by the type and concentration of dopants. It should be noted that pristine ε-FeSi is a narrow-gap semiconductor [31]; however, it can exhibit metallic-like behavior when doped or when present as a secondary phase due to impurity-induced metallization, and α-Fe_2_Si_5_ also exhibits metallic behavior. Therefore, they are unsuitable for thermoelectric applications due to their negative impact on the Seebeck coefficient (*S* = −∆*V*/∆*T*, where ∆*V* is the thermoelectric voltage, and ∆*T* is the temperature gradient across the material). In contrast, β-iron silicide (β-FeSi_2_) is a semiconductor with a bandgap of ~0.7 eV that has gained attention in TE applications.

**Figure 2.**
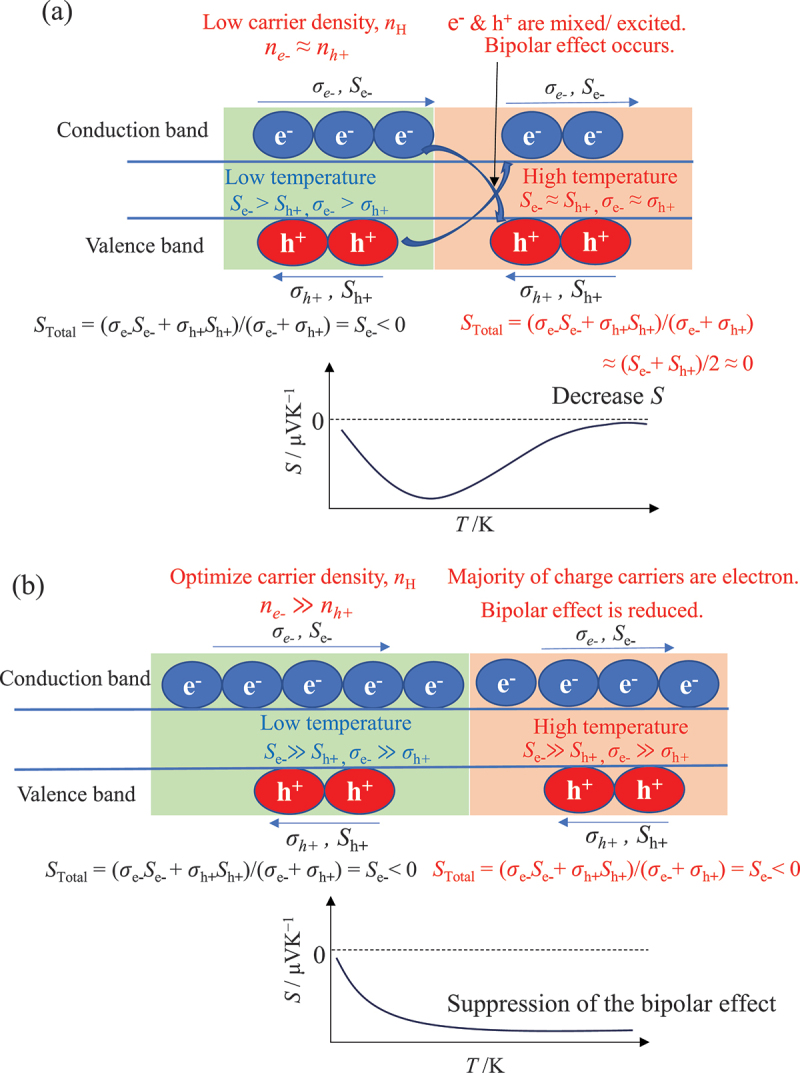
(a) A schematic explains the mechanism of the bipolar effect that occurs at high temperatures for low carrier density semiconductors, resulting in a significant reduction in Seebeck coefficient. (b) The increase in electron density contributes to the reduction in the bipolar effect at high temperatures, resulting in an improvement in the Seebeck coefficient.

It should be noted that the performance of pure β-FeSi_2_ is still limited due to its low carrier density (*n* ~10^16^ cm^−3^) and narrow bandgap (*E*_g_ ~0.7 eV) [32,33], leading to high electrical resistivity and bipolar effect, respectively. The bipolar effect has a negative impact on |*S*|. Figure 2(a) explains the phenomenon of how the bipolar effect at low *n* contributes to the reduction in |*S*|. The Seebeck effect arises from two types of charge carriers (electrons and holes) with opposite polarities. At elevated temperatures and low carrier density, their opposing contributions can cancel each other out, reducing the overall Seebeck effect, which is an undesirable outcome for thermoelectric applications [34–36]. These issues can be solved by increasing the carrier density of the system. Figure 2(b) explains the mechanism of reducing the bipolar effect by increasing the carrier density for n-type material. When the carrier density of electrons increases, the majority of charge carriers are electrons; hence, the |*S*| can be improved in high-temperature regions. The same concept is applied to p-type materials when the carrier density of holes is improved. Importantly, the enhancement of carrier density contributes not only to the reduction of the bipolar effect but also to the decrease in electrical resistivity, resulting in an improvement in power factor.

The carrier density of β-FeSi_2_ can be effectively enhanced by doping impurities into either Fe or Si sites. In addition, the conduction properties of β-FeSi_2_ can be tuned into both n-type and p-type. The increase in carrier density of electrons of n-type materials can be obtained by doping elements having more valence electrons into the Fe or Si site. On the other hand, the increase in carrier density of holes of p-type can be obtained by doping with elements having fewer valence electrons. For example, the n-type β-FeSi_2_ can be obtained by substituting Co or Ni into the Fe site, while the p-type ones can be achieved by doping Mn or Cr. The doping strategy could not only contribute to enhancing *PF* but also reduce thermal conductivity. A recent study found that cobalt (Co) dopant introduces the strain to β-FeSi_2_, leading to lattice softening. The lattice softening reduces phonon speed and consequently reduces the lattice thermal conductivity [37]. In addition, Qui et al. [38]. reported that Ir doping had a high solubility ~16% in n-type β-FeSi_2_, resulting in a significant improvement in *PF* values. Ir atoms also act as heavy elements to reduce the thermal conductivity of β-FeSi_2_ down to 2.8 Wm^−1^K^−1^ at room temperature. As a result, the maximum *ZT* of 0.6 at 1000 K was obtained by Ir doping. Such a *ZT* value is comparable to or even higher than other conventional TE materials, such as Heusler alloys [13,39] and oxide materials [40–42]. The addition of Co and Ni dopants also helps to improve the power factor, but it was found that the amount of impurity phase increases with increasing doping concentration [43]. This tendency was also observed in the samples doped with Mn [44], Al [45], and Cu [46]. In addition, while attempting to reduce the thermal conductivity by doping with heavy elements such as Ge [45] and Ru [47], the formation of the metallic phase also occurs. It is important to note that the presence of a secondary metallic phase has a negative impact on TE performance. When the dopants reach their solubility limit, the formation of secondary metallic phases (ε and α-phases) in β-FeSi_2_ is dominant. As a result, this limits the improvement in carrier density and deteriorates the |*S*|. To improve the dopant’s solubility, it is necessary to understand how the structure changes with the doping level and its relation to the electrical and thermoelectric properties.

In this article, we review recent progress in doping strategies, structural modifications, and property optimization of β-FeSi_2_, a non-toxic and low-cost thermoelectric material. We discuss the mechanisms of dopant incorporation, their influence on carrier concentration and mobility, and the resulting impact on thermoelectric performance. We also discuss current challenges, emerging approaches, and future research opportunities toward realizing high-performance β-FeSi_2_-based thermoelectric materials.

## 2. Structural transition of β-FeSi_2_-based materials

**Figure 3.**
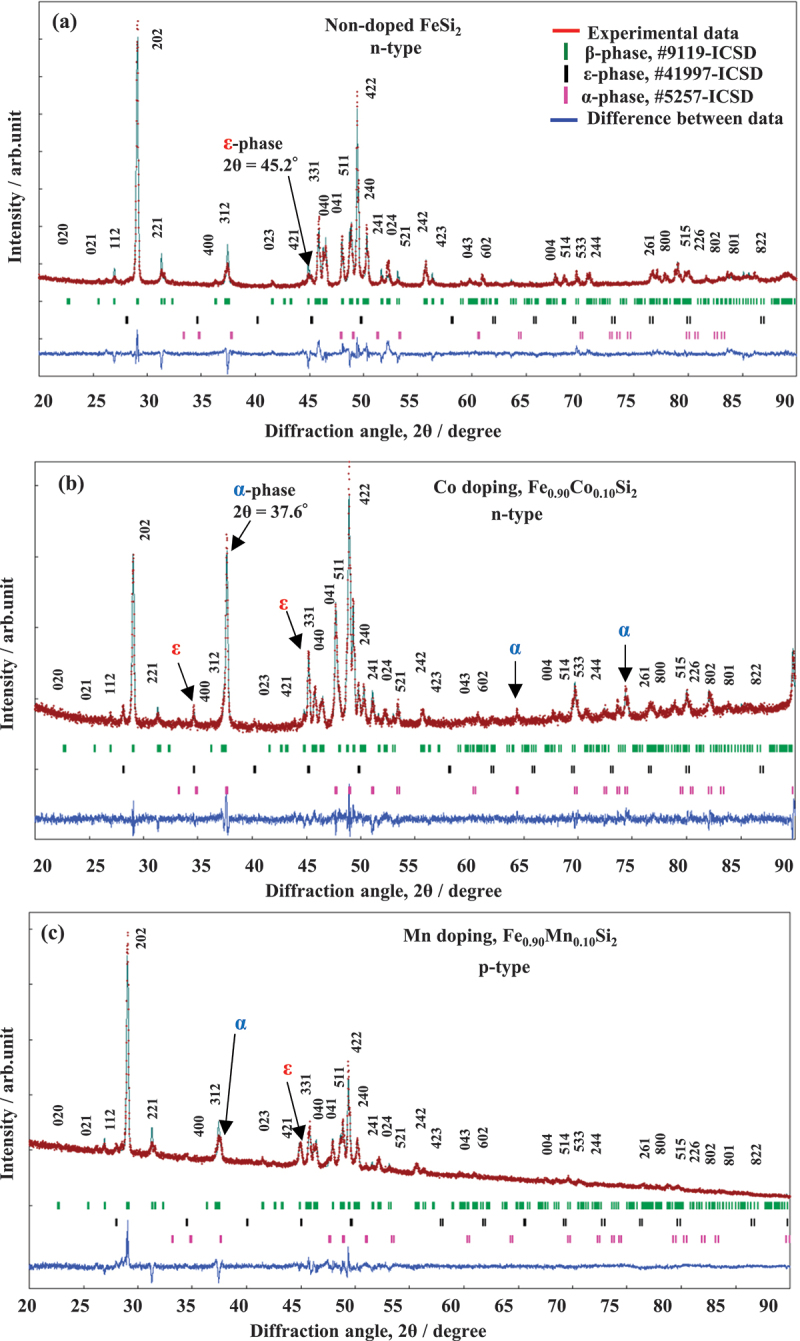
Rietveld refinement of the X-ray diffraction data of (a) non-doped FeSi_2_, (b) with 10%Co doping, and (c) with 10% Mn doping. The index peaks are the peaks of the semiconducting β-phase. The arrows indicate the peaks of impurity phases (ε and α-phases). Reproduced from the ref. [43] © *elsevier 2023*, and ref. [44] © *elsevier 2024.*

**Figure 4.**
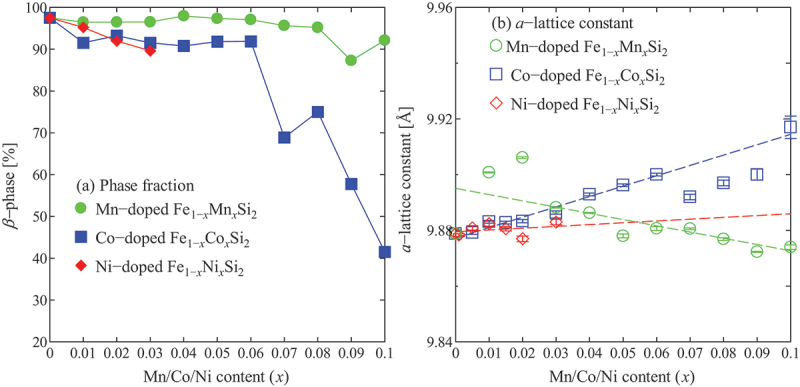
(a) The variation of β-phase and (b) *a*-lattice constant dependence of the doping levels of Mn, Co, and Ni obtained by the Rietveld analysis. The data plotted in this figure are collected from refs [43,44,51].

The X-ray diffraction (XRD) patterns are commonly used to identify the crystal structure of the fabricated materials [48,49], while the scanning electron microscope (SEM) is used to observe the variation in microstructures. The formation of secondary metallic phases (ε-FeSi and α-Fe_2_Si_5_) in β-FeSi_2_ is very sensitive to the dopants. The presence of secondary phases can be observed by both XRD and SEM. First, we discuss the observation of the presence of the metallic phase in β-FeSi_2_ using the XRD pattern. Figure 3 shows the result of Rietveld analysis from XRD data for non-doped FeSi_2_, 10% Co-doped, and 10% Mn-doped samples prepared by arc melting and heat treatment process [43,44]. In Figure 3(a) , the non-doped sample was crystallized in semiconducting β-phase, as confirmed with the indexed peaks. There is also a trace of ε-phase (at 2θ = 45.2°) as observed on the right side of the 421 peak. However, the intensity of this ε-phase is much lower than that of the sample with 10% Co and 10% Mn doping, as illustrated in Figure 3(c,d). In addition, it is shown that the α-phase (at 2θ = 37.6°) is also dominant with the addition of dopants Co and Mn. Therefore, the XRD patterns could tell us the presence of secondary phases, and their peak intensities increase with increasing dopants. Regarding the lattice parameters, it was reported that the lattice constants and volume tend to increase with increasing doping levels of Co [33,50], Mn [44], and Ir [38]. This is because when substituting the dopants into the β-FeSi_2_, the atomic size of Co (*r*_Co_ = 1.26 Å), Mn (*r*_Mn_ = 1.39 Å), and Ir (*r*_Ir_ = 1.37 Å) is larger than that of Fe (*r*_Fe_ = 1.25 Å). Nishida et al. mentioned that the increase in the lattice constants indicates that the Co atoms are dissolved in the solid solution of β-FeSi_2_ [50]. However, our research group found that even though the lattice constant keeps increasing up to 10% Co doping [33], the amount of β-phase remarkably drops to ~70% at a doping level of ≥7% Co [43]. The remarkable decrease in β-phase indicates that dopants reach their solubility limits in β-FeSi_2_. The degradation of the β-phase at various doping levels of Mn, Co, and Ni [43,44,51] is reported in Figure 4(a) . As shown in Figure 4(b) , the lattice constant of β-Fe_1-*x*_Mn_*x*_Si_2_ decreases with increasing *x*, while that of β-Fe_1-*x*_Co_*x*_Si_2_ and β-Fe_1-*x*_Ni_*x*_Si_2_ increases with increasing *x*. The trend appears not to be strictly monotonic. The deviation from the linear trend is thought to reflect effects such as solid solution limits within the β phase or precipitation of the ε phase or α phase. Such phase evolution can locally modify the lattice structure, leading to the observed non-monotonic behavior. In particular, the lattice constant of β-Fe_1-*x*_Co_*x*_Si_2_ deviates from the linear relationship at *x* > 0.06, strongly suggesting that the lattice constant is directly correlated with the solubility limit via Vegard’s law. The tendency indicates that the amount of β-phase in Mn-doped samples is more stable than that of Co and Ni, as the β-phase could be maintained at ~95% up to 8% Mn doping. This indicates that Mn is more soluble than Co and Ni in the β-FeSi_2_ system. In addition, Cheng et al. [52]. recently reported that by prolonging the annealing time up to 15 days at 1023 K, the single β-phase could be achieved up to 8% Co doping based on the observation of XRD pattern and SEM images. However, the quantification of the phase occupation was not provided. Therefore, it is necessary for every study to investigate the phase fraction when the dopants are introduced to β-FeSi_2_-based materials, as the formation of a secondary metallic phase negatively impacts the TE performance.

**Figure 5.**
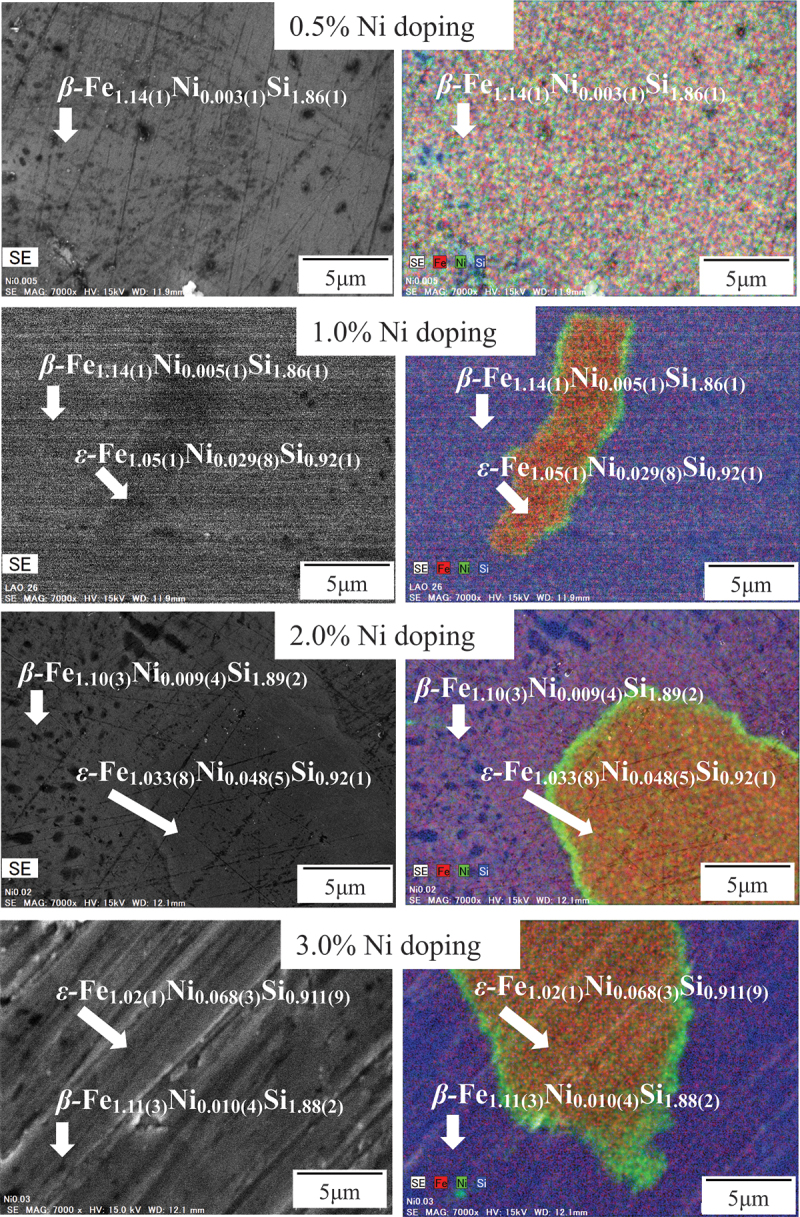
SEM-EDS mapping to observe the variation in microstructure of Ni-doped β-FeSi_2_. The Ni doping level is from 0.5% to 3%. The Fe elements are mapped in red, Ni in green, and Si in blue. Reproduced from ref. [53] © *2023 by the authors, licensed under cc by 4.0* (https://creativecommons.org/licenses/by/4.0/).

Next, we review how the microstructures of β-FeSi_2_ change with the addition of dopant. Our group has recently employed SEM-EDS techniques to investigate the effect of Ni doping on the microstructure of β-FeSi_2_ with varying Ni doping levels from 0.5% to 3.0% [53]. As illustrated in Figure 5, for all samples, in the area of the β-phase, the Fe atomic concentration is approximately 1/3, while that of Si is approximately 2/3. This indicates a Fe: Si ratio of about 1:2, corresponding to β-FeSi_2_. On the other hand, in the area of ε-phase, the Fe: Si ratio is about 1:1, corresponding to the secondary metallic phase of ε-FeSi. Notably, for 0.5% Ni doping, the microstructure shows a single β-phase, and the distribution of all elements is homogeneous, indicating that Ni is well soluble in β-FeSi_2_. However, the presence of ε-phase is found in ≥1.0% Ni-doping samples, and the grain size of ε-phase increases with the doping level. In addition, the richness of Fe and Ni elements is accumulated in the ε-phase. A similar tendency was observed in Mn-doped samples [44]. Moreover, even if the nominal doping level is 3%, the actual Ni concentration measured in the β-phase was about 1.0% only. This indicates the solid solution limit of Ni in β-FeSi_2_ is 1.0%. Due to its low solubility, the carrier density of Ni-doped samples is lower than that of other n-type materials, as listed in Table 1. As a result, the maximum *ZT* of only 0.019 is obtained in 0.5% Ni doping due to the small amount of impurity phase. However, such a low value is still far from practical applications. In addition, it was found that the β-phase linearly decreased with Ni addition [43], resulting in a decrease in TE performance. However, the optimization of annealing conditions (temperature and time) could help to maintain the β-phase while increasing the solid solution of the dopant. For example, based on a recent study [52], the microstructural observation showed that the grain of the ε-phase almost disappeared for 8% Co-doped β-FeSi_2_ after a long annealing time of 15 days and a lower temperature of 1023 K. More importantly, the microstructure analysis of Qiu et al. [38]. showed that there was no presence of any impurity phase at a high Ir doping level of 16% at an annealing time of 48 h and a temperature of 1173 K, resulting in a maximum *ZT* of 0.6 at 1000 K. On the other hand, Dąbrowski et al. [59]. investigated the variation of the microstructure of β-FeSi_2_ with various dopants, including Co, Mn, Al, and P. They found that the grain ε-phase appeared in Co-and Mn-doped samples but was absent in Al and P-doped ones. In addition, another study by their group showed that the grain of ε-phase increases when alloying with B_4_C particles [60]. Therefore, the formation of the impurity phase in the β-FeSi_2_ system is sensitive not only to the heat treatment condition but also to the type and concentration of dopants. The observation of microstructure transition is important to understand homogeneity, solubility, and the presence of secondary phase, which has a remarkable contribution towards optimizing the TE properties of silicide-based materials.

**Table 1.** Room temperature data of carrier density (*n*), mobility (*μ*), and calculated electrical resistivity (*ρ*) of n-type β-FeSi_2_ with various dopants.

Dopant	Conduction type	*n* [cm^−3^]	*μ* [cm^2^V^−1^s^−1^]	*ρ* [Ωcm]	Reference
Non-doped	n	2.3 × 10^16^	37	7.33 × 10°	Sam et al. [33].
1% Co	n	1.8 × 10^19^	5.9	5.88 × 10^−2^	
3% Co	n	1.2 × 10^20^	3.5	1.49 × 10^−2^	
6% Co	n	4.2 × 10^20^	1.9	7.82 × 10^−3^	
1% Co	n	2.3 × 10^20^	0.50	5.43 × 10^−2^	Tani et al. [54]
3% Co	n	8.1 × 10^20^	0.39	1.98 × 10^−2^	
4% Co	n	1.9 × 10^21^	0.4	8.21 × 10^−3^	Cheng et al. [52].
8% Co	n	4.0 × 10^21^	0.3	5.20 × 10^−3^	
12% Co	n	1.1 × 10^22^	0.3	1.89 × 10^−3^	
16% Co	n	1.5 × 10^22^	0.2	2.08 × 10^−3^	
2.9% Co	n	7.6 × 10^20^	0.26	3.16 × 10^−2^	Birkholz et al. [55]
5% Co	n	1.9 × 10^21^	0.35	9.39 × 10^−3^	Nishida et al. [29]
3% Co	n	1.6 × 10^18^	6.0	6.50 × 10^−1^	Brehme et al. [56].
3%Co + 1%Ni	n	1.4 × 10^19^	25	1.78 × 10^−2^	Sam et al. [57].
1% Ni	n	1.9 × 10^19^	0.53	6.20 × 10^−1^	Tani et al. [54]
3% Ni	n	4.7 × 10^19^	0.27	4.92 × 10^−1^	
1% Pt	n	4.8 × 10^20^	0.65	2.04 × 10^−2^	Tani et al. [58].
3% Pt	n	9.2 × 10^20^	0.66	1.04 × 10^−2^	
5% Pt	n	9.2 × 10^20^	0.73	7.78 × 10^−3^	
16% Ir	n	1.1 × 10^22^	0.7	8.11 × 10^−4^	Qiu et al. [38].

The formation of secondary phases, such as ε- and α-phases, is sensitive to the preparation route, and they can readily form during high-temperature processes like arc melting. For non-doped samples, these secondary phases can be converted to the β-phase ~97% through appropriate annealing, as confirmed by Rietveld refinement [43]. Dopants also influence secondary-phase formation differently. To assess the influence of annealing in spark plasma sintering (SPS) samples, Cheng et al. [52]. investigated Co-doped β-FeSi_2_ prepared under the same SPS conditions. They observed that secondary phases were formed in 8% Co-doped sample by SEM. By increasing the annealing time from 5 to 15 days and lowering the temperature from 900°C to 750°C, the secondary phases disappeared. These observations indicate that for both arc-melted and SPS-prepared samples, annealing is crucial to obtain a high-purity β-phase. Moreover, higher doping levels require optimized annealing conditions to suppress secondary-phase formation.

## 3. Electrical transport improvement of β-FeSi_2_-based materials

The conduction properties of β-FeSi_2_ can be tuned to an n-type or p-type depending on the dopants. If Fe or Si is substituted with elements having fewer valence electrons (for example, Mn to the Fe site or Al to the Si site), the electrical conduction is p-type. In contrast, if they are substituted with elements having more valence electrons, the materials are changed to n-type. From the literature Tables 1 and 2, respectively, list the electrical properties of n-type and p-type β-FeSi_2_-based materials, including carrier density (*n*), mobility (*μ*), and the calculated electrical resistivity (*ρ* = |*e*|^−1^*n*^−1^*μ*^−1^, where *e* is the elementary charge constant). In Table 1, for n-type, Sam et al. [33] and Tani et al. [54] reported that the carrier density of Co-doped samples is about four orders of magnitude higher than that of pure β-FeSi_2_. As a result, the *ρ* can be effectively reduced from ~10° to ~10^−2^ − 10^−3^ Ωcm, as the doping level increases from 0% to 6%. Importantly, Cheng et al. [52] attempted to increase the solid solution limits of Co atoms by prolonging the annealing time for 15 days. They obtained a high carrier density up to ~10^22^ cm^−3^ with solubility of Co up to 16%. On the other hand, by adding co-doping (Co + Ni), the mobility can be improved to 25 cm^2^V^−1^s^−1^ because the substitution of Ni for Co probably modifies the electronic band structure, leading to a lighter effective mass [57]. Tani et al. found that, at the same doping level, the carrier density of Ni-doped samples is lower than that of Co-doped samples [54]. This is because Co atoms have a higher solubility in the β-FeSi_2_ system than Ni atoms. The Pt element can also be used to tune the electrical properties of n-type β-FeSi_2_. Importantly, the lowest electrical resistivity of ~10^−4^ Ωcm was obtained by 16% Ir doping [38]. This exceptional heavy doping led to a remarkable improvement in the *ZT* value of 0.6 due to the improvement of the power factor. Table 2 summarizes the room temperature electrical properties of p-type β-FeSi_2_ with Mn doping on the Fe site and Al doping on the Si site. The carrier density of Mn-doped samples can be enhanced to ~10^18^ − 10^19^ cm^−3^ as the doping level increases from 1% to 10% [44,51]. However, the higher carrier density of ~10^20^ cm^−3^ could be obtained by Al doping as reported by Du et al. [61]. Therefore, doping with Al is more effective in improving the carrier density and electrical conductivity for β-FeSi_2_.

**Table 2.** Room temperature data of carrier density (*n*), mobility (*μ*), and calculated electrical resistivity (*ρ*) of p-type β-FeSi_2_ with some dopants.

Dopant	Conduction type	*n* [cm^−3^]	*μ* [cm^2^V^−1^s^−1^]	*ρ* [Ωcm]	Reference
Non-doped	p	4.5 × 10^17^	0.9	1.54 × 10^1^	Tani et al. [54]
1% Mn	p	2.9 × 10^18^	5.6	3.84 × 10^−1^	Sam et al. [51].
3% Mn	p	6.1 × 10^18^	4.3	2.38 × 10^−1^	
5% Mn	p	1.8 × 10^19^	3.3	1.05 × 10^−1^	Sam et al. [44].
7% Mn	p	4.4 × 10^19^	2.6	5.46 × 10^−2^	
10% Mn	p	2.0 × 10^19^	2.0	1.56 × 10^−1^	
2% Al	p	2.0 × 10^18^	2.0	1.56 × 10^0^	Birkholz et al. [55]
1% Al	p	5.0 × 10^19^	8.5	1.47 × 10^−2^	Du et al. [61]
1.5% Al	p	1.2 × 10^20^	9.0	5.78 × 10^−3^	
2% Al	p	1.6 × 10^20^	8.0	4.88 × 10^−3^	

It should be noted that for both n-type and p-type, mobility has an inverse tendency with carrier density or doping level. This relationship can be explained by the single parabolic band-acoustic phonon scattering model (SPB – APS model), which is commonly used to describe carrier transport in thermoelectric semiconductors. For example, the mobility decreases from 37 cm^2^V^−1^s^−1^ to 1.9 cm^2^V^−1^s^−1^ as Co doping increases from 0% to 6% [33]. A similar tendency was also observed in Ni-doped samples as reported by Tani et al. [54]. The increase in both carrier density and mobility contributes to reducing electrical resistivity, leading to the enhancement of TE performance. A recent work attempted to improve the mobility of the Co-doped sample with Ni substitution [57]. Although the mobility was improved to 25 cm^2^V^−1^s^−1^ with the addition of Ni, the Seebeck coefficient degraded due to the increase in the metallic phase. Therefore, the investigation of the phase fraction and microstructure evolutions is also important to understand the dopants’ solubility, as it is related to improvement in carrier density and mobility.

## 4. Thermoelectric properties of β-FeSi_2_-based materials

### 4.1. Power factor improvement strategy

**Figure 6.**
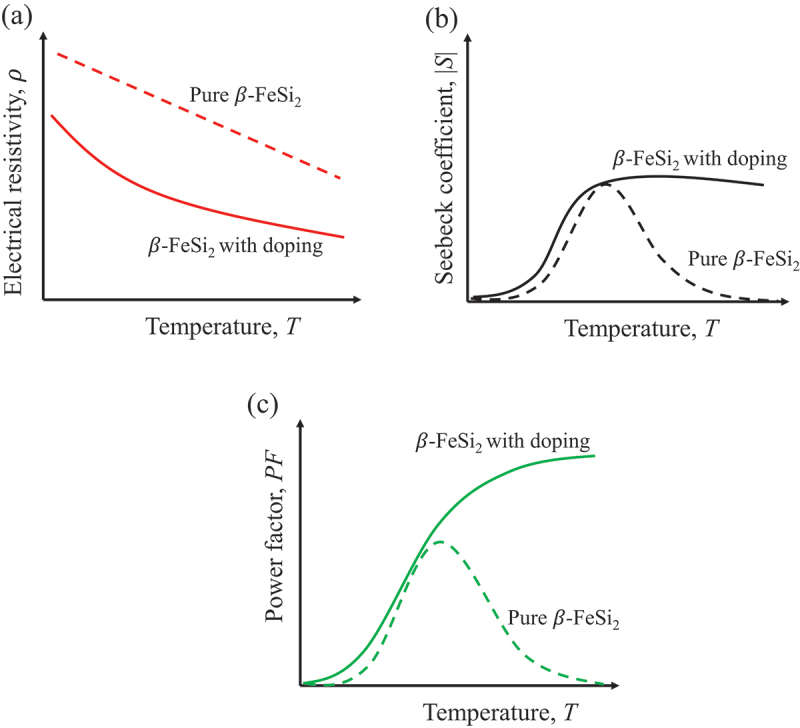
Schematic illustration of the transport properties improvement of *β*-FeSi_2_ with doping. (a) Decrease in electrical resistivity (*ρ*), (b) improvement of Seebeck coefficient (*S*) at high temperature region, and (c) enhancement in power factor (*pf* = *S*^2^*ρ*^−1^).

Power factor is one of the key parameters proportional to *ZT* value. The power factor is defined by: *PF* = *S*^2^*ρ*^−1^, where *S* is the Seebeck coefficient and *ρ* is the electrical resistivity. To improve the *PF* value, it is necessary to decrease *ρ* and increase *S*. Figure 6 illustrates the typical mechanism of transport properties improvement in β-FeSi_2_ with the addition of dopants. Due to low carrier density (~10^16^ − 10^17^ cm^−3^) [33,54], the *ρ* value of pure β-FeSi_2_ is higher than that of other conventional TE materials. The reduction in *ρ* can be effectively obtained by increasing the carrier density with elemental doping (Figure 6(a)). Doping not only decreases *ρ* but also stabilizes *S* in the high-temperature region due to the reduction of bipolar effect. Figure 6(b) shows the schematic of improving *S* by the doping approach. As mentioned in the Introduction section (Figure 2), the narrow bandgap and low carrier density cause the decrease in *S* at high temperatures because of bipolar diffusion. This bipolar effect can be reduced by increasing the carrier density with doping. Therefore, doping can simultaneously improve *S* and decrease *ρ*. As a result, as shown in Figure 6(c), the thermoelectric power factor of β-FeSi_2_ is remarkably improved via the doping technique.

Although doping significantly improves the *PF*, the doping levels should be optimized. There are two problems to be considered, such as solution limits and the trade-off relationship between carrier density and Seebeck coefficient according to Mott’s theory. When the dopants reach their solubility limits, the secondary metallic phases form, and the Seebeck coefficient consequently decreases [51]. In addition, the inverse relation between the carrier density and the Seebeck coefficient is defined by Mott’s formula [62]:

(1) $$|S|=\frac{k_{B}^{2}T}{3\left|e\right|\hbar^{2}}m*{\left(\frac{\pi }{3n}\right)}^{2/3}$$

where |*S*| is the absolute Seebeck coefficient, *k*_*B*_ is the Boltzmann constant, *T* is the temperature, *ℏ* is the Planck constant, *e* is the elementary charge, m* is the effective mass, and *n* is the carrier density. From Equation (1), if the carrier density increases too high due to high doping levels, the Seebeck coefficient decreases. Based on the theoretical calculation reported by Pandey et al. [63], the optimum carrier density for enhancing β-FeSi_2_ performance is in the range from 3 × 10^20^ to 2 × 10^21^ cm^−3^. However, experimental optimization of the doping level is also necessary for improving the power factor of β-FeSi_2_.

**Figure 7.**
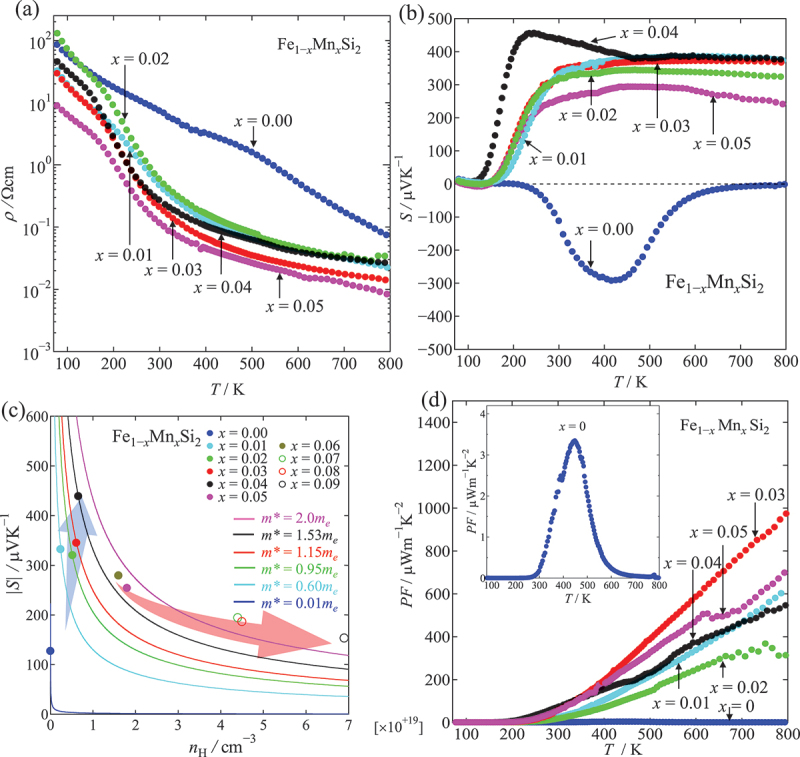
Transport properties of β-Fe_1−*x*_Mn_*x*_Si_2_ (0 ≤ *x* ≤ 0.05). (a) Temperature dependence of electrical resistivity *ρ*, and (b) Seebeck coefficient *S*. (c) Seebeck coefficient vs. carrier density at room temperature. The solid lines are the calculated data from the Mott formula in Equation (1) at various effective masses m* = *xm*_e_, where *x* is a variable and *m*_e_ is the static mass of the electron, i.e. 9.10938 × 10^−31^ kg. The blue arrow indicates that the Seebeck coefficient increases with doping level due to the increase in effective mass, while the red arrow indicates the Seebeck coefficient decreases with increasing carrier density. (d) Temperature dependence of power factor *PF*. Reproduced from ref [51]. © *elsevier 2024.*

An experimental study of the optimization of the Mn doping level for p-type β-FeSi_2_ has been conducted [51]. Based on the fraction analysis by Reitveld refinement, the amount of β-phase could be maintained at 95% for Mn doping levels of ≤8%. However, the elemental mapping analysis by SEM-EDS indicates that the Mn reaches its solid solution limit in the β-phase at 5% Mn doping [44]. This suggests that the optimum Mn doping level should be lower than 5%. Figure 7 illustrates the variation in the transport properties of β-Fe_1−*x*_Mn_*x*_Si_2_ (0 ≤ *x* ≤ 0.05). In Figure 7(a), the electrical resistivity of all samples decreases with increasing temperature, indicating the semiconducting behavior over the measured temperature range from 80 K to 800 K. Notably, the resistivity of Mn-doped samples is about two orders of magnitude lower than that of the non-doped sample, and the resistivity continues to decrease with Mn doping level. As for the Seebeck coefficient, Mn doping tunes the conduction properties of β-FeSi_2_ from n-type to p-type (Figure 7(b)). The Seebeck coefficient for the *x* = 0 sample decreases with increasing temperature in the high-temperature region due to the bipolar diffusion effect. With the addition of Mn, the Seebeck coefficient is improved due to the suppression of the bipolar effect when the carrier density is optimized. However, for *x* ≥ 0.05, the Seebeck coefficient decreases with increasing *x* because of the increased carrier density, as explained by Mott’s theory. The relationship between the Seebeck coefficient, carrier density, and effective mass can be understood from Figure 7(c). For 0 ≤ *x* ≤ 0.04, the Seebeck coefficient increases with increasing doping level due to the increase in effective mass m* from 0.01 *m*_e_ to 1.53 *m*_e_. The m* was estimated using the Mott relation under the parabolic-band approximation, which is equivalent to the single parabolic band (SPB) model. However, the large variation in m* suggests that this simplified model may not fully capture the non-parabolic and multivalley nature of Mn-doped β-FeSi_2_. On the other hand, for *x* ≥ 0.04, as the carrier density increases, the Seebeck coefficient then decreases with doping level. As a result, the maximum power factor *PF* = 970 μWm^−1^K^−2^ at 800 K was obtained at the optimum doping level of 3% Mn, as shown in Figure 7(d). Such a value is much higher than that of the non-doped sample (*PF* = 3.4 μWm^−1^K^−2^). Du et al. [61]. investigated another p-type material by Al doping on the Si site of β-FeSi_2-*x*_Al_*x*_ (*x* = 0.02, 0.03, and 0.04). Similar to the Mn-doped samples, the Seebeck coefficient of Al-doped samples was improved, and the electrical resistivity was effectively reduced with Al doping levels. As a result, the highest *PF* of 1100 μWm^−1^K^−2^ at 500 K − 600 K was obtained in an Al doping level of *x* = 0.04 [61]. Komabayashi et al. [64]. reported that the improvement in *PF* can also be obtained by Cr and V doping, with the *PF* value of 260 μWm^−1^K^−2^ and 230 μWm^−1^K^−2^ at room temperature, respectively.

For n-type β-FeSi_2_, the improvement in the *PF* can be obtained by doping with Pt [58,64], Pd [64], Ni [53], Co [52,65–67], and Ir [38]. For example, at room temperature, the addition of Pt and Pd contributes to the *PF* enhancement of 190 μWm^−1^K^−2^ and 490 μWm^−1^K^−2^, respectively [64]. The addition of Ir also improves *PF* = 1800 μWm^−1^K^−2^ at 1000 K. This improvement is mainly due to the reduction of electrical resistivity. Recently, our group investigated the effects of Ni doping (0.5% − 3.0%) on the TE properties of β-FeSi_2,_ and the maximum *PF* of 200 μWm^−1^K^−2^ was obtained at a very low doping concentration of 0.5% Ni [53]. This indicates that Ni has a low solubility in β-FeSi_2_. In addition, Tani et al. reported the TE properties of Co-doped samples, where the doping levels are from 1% to 10%. They obtained the highest *PF* value of 1100 μWm^−1^K^−2^ in the 5% Co-doped sample. This value is in agreement with that reported by Birkholz et al. [55]. However, the *PF* value decreased when increasing Co doping up to 10% due to a reduction in the Seebeck coefficient. The reduction in the Seebeck coefficient could probably be caused by its inverse relationship with the carrier density or due to the increase in the metallic phase. To solve this problem, Cheng et al. [52]. attempted to improve the solubility of Co by optimizing the annealing conditions. As a result, they obtained a high solubility of 8% Co in β-FeSi_2_ by increasing the annealing time to 15 days. Such a high solubility contributed to the *PF* enhancement of 1400 μWm^−1^K^−2^ at 900 K. This indicates that optimizing the annealing condition improves the solid solution of dopants, contributing to *PF* enhancement. It should be noted that different dopants contribute to different levels of *PF* improvement. This is because of their solid solution limit levels, while improving the solubility of dopants significantly improves the *PF* value of the β-FeSi_2_-based materials.

### 4.2. Thermal conductivity reduction strategy

**Figure 8.**
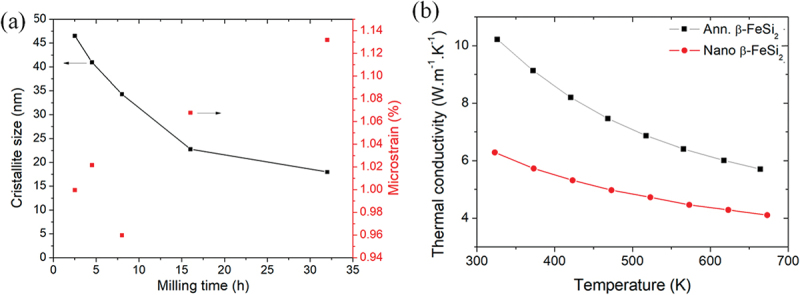
The result of thermal conductivity reduction by nanostructuring with the milling process. (a) The crystallite size as a function of ball milling time (left axis) and micro-strain on the right axis. (b) The temperature dependence of the thermal conductivity of annealed β-FeSi_2_ and nano β-FeSi_2_ (fabricated by ball milling techniques). Reproduced from ref [68]. *© 2021 by the authors, licensed under cc by 4.0* (https://creativecommons.org/licenses/by/4.0/).

The reduction in thermal conductivity is necessary because it has an inverse relation to the *ZT* value. The total thermal conductivity is dominated by the lattice and electronic part (*κ*_total_ = *κ*_lattice_ + *κ*_electronic_). In semiconducting β-FeSi_2_, the *κ*_lattice_ is more dominant than *κ*_electronic_, where major of the heat is transported by phonons. Therefore, the *κ*_total_ of β-FeSi_2_ can be reduced by nanostructuring techniques and doping with heavy elements. Abbassi et al. [68]. attempted to reduce the *κ*_total_ using nanostructures by the milling process. As shown in Figure 8(a), they found that the crystallite size decreases with increasing milling time, while the microstrain increases. As a result, the thermal conductivity of nano β-FeSi_2_ (fabricated by ball milling techniques) is remarkably lower than the annealed one, as shown in Figure 8(b). The reduction of thermal conductivity upon ball milling is primarily attributed to enhanced phonon scattering at grain boundaries, which effectively suppresses the transport of heat-carrying phonons. The ball milling process refines the grains, increases the density of grain boundaries, and introduces additional structural defects, all of which act as strong phonon scattering centers. Although minor lattice disorder (lattice softening) may also occur, its contribution is secondary compared to grain boundary scattering. The relationship between *κ*_lattice_, *τ*, and *υ*_g_ can be expressed by: *κ*_lattice_ = (1/3) *C*_v_*υ*_g_^2^*τ* = (1/3) *C*_*v*_*υ*_*g*_*l*, where *C*_v_ is the specific heat at constant volume, and *l* is the phonon mean free path [3]. Therefore, the decrease in *τ* due to phonon scattering at small crystallite size and the decrease in *υ*_g_ due to lattice softening caused by strain contribute to the reduction in *κ*_lattice_ and *κ*_total_. In addition, Le Tonquess et al. [69]. introduced the stacking fault into pure β-FeSi_2_ and Co-doped β-FeSi_2_ synthesized by the magnesioreduction (MR) process. They found that the stacking probabilities of MR samples (*SF* = 10.7(2) % for pure β-FeSi_2_ and *SF* = 10.4(1) % for Co-doped β-FeSi_2_) were higher than that of arc-melted samples (*SF* = 3.7(1) % for pure β-FeSi_2_ and *SF* = 3.2(1) % for Co-doped β-FeSi_2_). As a result, the *κ*_lattice_ of the pure sample and the Co-doped sample, respectively, decreases by 24% and 17% compared to that of the arc-melted samples. This reduction is attributed to the enhanced phonon scattering at the stacking faults. However, the electrical resistivity also increases in MR samples. This could be because of the decrease in mobility when electrons scatter more frequently at the stacking fault. In addition, Isoda et al. [15]. investigated the effect of grain size on the *κ*_total_ for both p-type Mn-doped and n-type Co-doped β-FeSi_2_ prepared by the ball milling process. For both p-type and n-type samples, the *κ*_total_ and with decreasing grain size from 24 μm to 11 μm and from 10 μm to 7 μm, respectively. This is mainly because of the decrease in *κ*_lattice_, while the phonons are scattered by the grain boundaries. However, the electrical resistivity in the p-type sample increases with decreasing grain size (more boundaries are formed), which has a trade-off relationship with *κ*_total_. This phenomenon is similar when the stacking faults were introduced to β-FeSi_2_ [69,70]. This indicates that the optimization of grain size, stacking fault, and crystallite size is necessary to selectively scatter the phonons while preserving the electron transport. This is about balancing the characteristic size of the structure in relation to the phonon and electron mean free path (MFP). It was reported that the non-doped β-FeSi_2_ thin film and nanowire have the MFP of several hundred nanometers [71,72], while the electron MFP of ferromagnetic material (FeSi_2_ can be ferromagnetic at the nanoscale) is only several nanometers [73,74]. However, the MFP values of phonon and electron at the bulk scale or when the dopants are added, can be different from those in the non-doped or thin film structures. Therefore, this is the opportunity for future research to conduct experimental or theoretical studies on the variation of electron and phonon MFP in the metal-doped β-FeSi_2_ system. This could help us to understand the electro-thermal transport behavior and to design the characteristic sizes of the structure to balance the trade-off relationship between electrical and thermal conductivity toward enhancing the performance of the materials.

**Figure 9.**
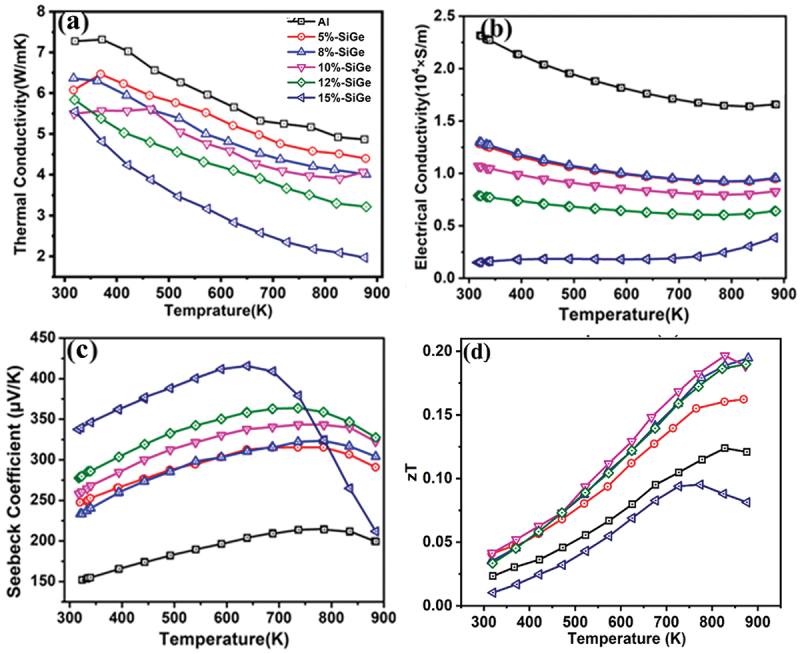
Improved thermoelectric performance by reducing thermal conductivity of Al-doped β-FeSi_2_ with the addition of Ge as a heavy element. (a) Total thermal conductivity, (b) electrical conductivity, (c) Seebeck coefficient, and (d) *ZT* values with temperature dependence. Reproduced from ref [45]. *© 2025 by the authors, under the cc by license* (http://creativecommons.org/licenses/by/4.0/).

Another strategy to reduce the *κ*_total_ of the β-FeSi_2_ system is the introduction of heavy elements such as Ge [45,75,76], Ru [47], Os [61], and Ir [38]. For example, Sangwan et al. [45]. investigated the effect of SiGe addition on the TE properties of Al-doped β-FeSi_2_ (Figure 9). The *κ*_total_ values remarkably decrease with increasing SiGe concentration (Figure 9(a)). In this case, systems containing heavier element (Ge) is anticipated to have reduced thermal conductivity, since larger atomic mass generally leads to lower phonon frequencies, stronger optical – acoustic scattering, and slower sound velocities [77]. However, as shown in Figure 9(b), the electrical conductivity also decreases because the introduction of heavier elements enhances electron – phonon scattering and increases the effective mass of charge carriers, which reduces their mobility. In addition, modifications in the band structure caused by heavier atoms further limit carrier transport, resulting in lower conductivity. In Figure 9(c), the Seebeck coefficient is improved because heavier elements increase the effective mass of charge carriers and modify the band structure, which enhances the energy dependence of carrier transport. In addition, stronger scattering effects could favor high-energy carriers, further boosting the Seebeck coefficient. However, when the addition of SiGe is higher than 12%, the Seebeck coefficient remarkably decreases at high temperature. This is probably because the metallic ε-phase increases with increasing Ge concentration, as the formation of the metallic phase in the β-FeSi_2_ system is sensitive to dopant concentration. Prolonging the annealing time could contribute to maintaining the β-phase [52] and thus improving the Seebeck coefficient. As for *ZT* values (Figure 9(d)), the maximum value of 0.19 was obtained in the 10% SiGe-added sample, which is higher than the non-SiGe-added sample (*ZT* ~0.15). Moreover, Du et al. [47]. examined the effect of Ru incorporation as a heavy element on the thermoelectric properties of n-type Co-doped β-FeSi_2_. They found that Ru atoms are unevenly distributed, since their diffusion is limited during annealing at 1173 K. However, Ru doping significantly lowers the lattice *κ*_lattice_ by introducing mass and strain field fluctuations that scatter phonons, while its influence on electrical transport remains minor. As a result, a maximum *ZT* of 0.33 was achieved at 900 K in the 5% Ru-doped sample, representing a 27% improvement over the sample without Ru. They further attempted to reduce the thermal conductivity of p-type Al-doped β-FeSi_2_ with Os substitution [61]. At an Os concentration of 20%, the *κ*_lattice_ reached a minimum of 2.6 Wm^−1^K^−1^ at 900 K. Importantly, Os addition caused only a minor reduction in the *PF*, leading to a maximum *ZT* of 0.35 at 850 K. Recently, Qiu et al. [38]. reported that 16% Ir doping induces multi-valley electronic conduction and enhances phonon-electron scattering, resulting in a *ZT* of 0.6 at 1000 K. In the three studies above, the authors limited the Ru, Os, and Ir concentrations to 5%, 20%, and 16%, respectively, yet the maximum *ZT* values were achieved at these highest concentrations. This suggests that exploring higher Ru, Os, and Ir content is possible to achieve greater *ZT* values.

Tables 3 and 4, respectively, summarize the *ZT*_max_ values of n-type and p-type β-FeSi_2_ with various dopants using different fabrication techniques. The tables are listed in the order from low to high values of *ZT*_max_. It should be noted that the conduction behavior of the non-doped β-FeSi_2_ can be n-type or p-type depending on the purity of the raw elements, i.e. Fe and Si. When the sample was prepared with 5N-purity of raw elements, it could be n-type materials. On the other hand, 4N-purity raw elements could produce p-type materials [15]. However, as shown in Tables 3 and 4, the *ZT*_max_ ~0.01 of both n-type and p-type samples is very low compared to those with additions of dopants. For example, for n-type β-FeSi_2_-based materials, the maximum *ZT* of 0.3 and 0.6 can be obtained by Co and Ir doping, respectively (Table 3). Some oxide particles (SiO_2_, TiO, and Y_2_O_3_) [80,82] and heavy elements (Ge and Ru) [45,47,75,76,78] were added to the Co-doped β-FeSi_2_ to further reduce the *κ*_lattice_. Therefore, the *ZT* value can be enhanced due to the simultaneous improvement of *PF* and reduction in *κ*. For p-type β-FeSi_2_-based materials, the maximum *ZT* of 0.21 and 0.35 can be obtained by Mn and Al+Os doping, respectively (Table 4). It is noted that the *ZT*_max_ value of 0.35 for p-type is still lower than that for n-type (~0.6). The lower *ZT* values are primarily due to the low solid solution limit of dopants. Therefore, improving dopant solubility could further improve the *ZT* values.

**Table 3.** Summary of *ZT*_max_ values of n-type β-FeSi_2_ with various dopants using different processing methods. The order is classified from the low to high values of *ZT*_max_.

Dopant	Processing methods	*ZT*_max_	Temperature	References
Non-doped	Hot pressing (HP)	0.01	657 K	Kim et al. [78]
0.1% Ni	Arc melting	0.019	600 K	Sam et al. [53]
5% P	Ball milling/mechanical alloying (MA) + Pulse plasma sintering (PPS)	0.02	673 K	Dabrowski et al. [59]
2% P	MA+HP	0.033	672 K	Ito et al. [79].
2%Co + 2%Y_2_O_3_	MA+HP	0.058	650 K	Ito et al. [80].
2% Co	MA+HP	0.06	600 K	Ur et al. [81]
2%Co + 4%TiO	MA+HP	0.065	650 K	Ito et al. [82].
2%Co + 4%SiO_2_	MA+HP	0.078	650 K	Ito et al. [82].
1.77% Co	MA+HP	0.085	873 K	Redzuan et al. [83]
3% Co	Arc melting	0.099	720 K	Sam et al. [33].
5% Co + 4.2% Ge	HP	0.11	845 K	Kim et al. [78]
3% Pt	Spark plasma sintering (SPS)	0.14	847 K	Tani et al. [58]
3% Co	MA+PPS	0.15	773 K	Dabrowski et al. [59]
5% Co	MA+HP	0.182	923 K	He et al. [84].
5% Co	Gas-atomized sintering	0.22	900 K	Kimura et al. [85]
5% Co	SPS	0.25	940 K	Tani et al. [65]
6% Co	Rapid solidification +HP	0.25	908 K	Chen et al. [86]
8% Co	SPS	0.3	900 K	Cheng et al. [52]
3% Co + 1% Ni	Arc melting	0.31	720 K	Sam et al. [57].
6% Co + 5% Ru	SPS	0.33	900 K	Du et al. [47]
16% Ir	SPS	0.6	1000 K	Qui et al. [38]

**Table 4.** Summary of *ZT*_max_ values of p-type β-FeSi_2_ with various dopants using different processing methods. The order is classified from the low to high values of *ZT*_max_.

Dopant	Processing methods	*ZT*_max_	Temperature	References
Non-doped	Ball milling/mechanical alloying (MA) + Pulse plasma sintering (PPS)	0.01	673 K	Dabrowski et al. [59]
5% Cr	Hot pressing (HP)	0.03	657 K	Kim et al. [78]
24% Zr	MA+HP	0.03	1070 K	Ito et al. [12].
7% Al	MA+PSS	0.06	773 K	Dabrowski et al. [59]
8% Mn	MA+PSS	0.06	773 K	Dabrowski et al. [59]
5% Mn	Arc melting	0.07	800 K	Sam et al. [44].
3% Al	MA+HP	0.08	873 K	He et al. [84].
5% Mn	Gas-atomized sintering	0.10	900 K	Kimura et al. [85]
3% Mn	Arc melting	0.12	800 K	Sam et al. [51].
2% Al	Spark plasma sintering (SPS)	0.18	850 K	Du et al. [61]
10% Mn	SPS	0.21	1040 K	Tani et al. [87]
2% Al + 20% Os	SPS	0.35	850 K	Du et al. [61]

Regarding the band structure, Pandey et al. [63]. reported that the n-type β-FeSi_2_ outperforms the p-type due to fundamental differences in their electronic structures. The conduction band comprises multiple heavy, anisotropic pockets that provide a high density of states favorable for thermoelectric performance, while the valence band contains a light band near the VBM that limits the p-type thermopower. This band structure difference allows n-type doping to maintain high thermopower ( >200 μVK^−1^) over a wider carrier concentration range (3 × 10^20^ to 2 × 10^22^ cm^−3^) with reduced susceptibility to bipolar conduction effects. These computational insights explain the experimental observations of higher *ZT* in n-type β-FeSi_2_ and provide guidance for optimizing doping strategies.

**Figure 10.**
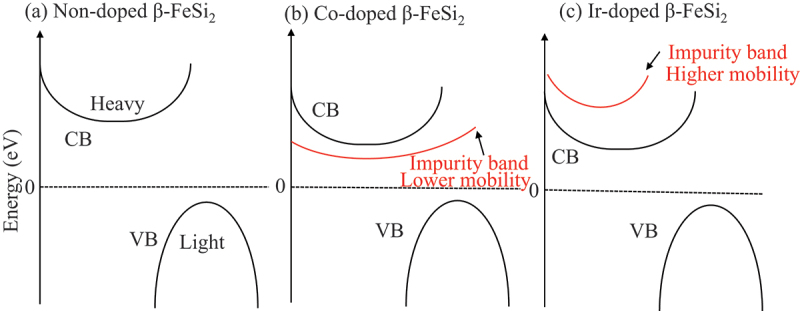
Schematic of the band structure of (a) non-doped, (b) Co-doped, and (c) Ir-doped β-FeSi_2_.

The schematic of the band structure of non-doped, Co-doped, and Ir-doped β-FeSi_2_ is illustrated in Figure 10. The computational study predicted superior n-type performance in β-FeSi_2_ at high carrier concentrations because the curvature in the conduction band is flatter, leading to a higher effective mass (Figure 10(a)) [63]. However, a recent experimental work by Cheng et al. [52]. reveals that the choice of dopant determines whether this potential is realized. The superior n-type performance stems from the favorable conduction band structure featuring multiple heavy valleys that provide a high density of states, whereas p-type performance is limited by a light valence band that reduces thermopower at practical doping concentrations [63]. Heavy Co doping up to 24% was achieved, confirming the feasibility of high n-type carrier concentrations. However, the maximum *ZT* of 0.3 occurred at only 8% doping and did not improve with further Co addition. This contrasts sharply with Ir-doped β-FeSi_2_, which achieved *ZT* = 0.6 at 16% doping as reported by Qiu et al. [38]. The disparity stems from fundamental differences in how Co and Ir modify the electronic structure. Co introduces localized impurity bands below the conduction band minimum, leading to low-mobility hopping conduction at low doping and impurity band conduction at high doping (Figure 10(b)). In contrast, Ir creates impurity bands above the CBM (Figure 10(c)), preserving the beneficial multi-valley conduction character predicted by Pandey et al. [63]. This explains why merely achieving high carrier concentration is insufficient, as the dopant must also maintain or enhance access to the favorable intrinsic conduction bands for optimal thermoelectric performance.

It should be noted that the improvement in thermoelectric performance of β-FeSi_2_-based materials is not solely due to carrier density optimization. Qiu et al. [38]. reported that Ir doping markedly reduces the total thermal conductivity to 2.8 Wm^−1^K^−1^ for β-Fe_0.84_Ir_0.16_Si_2_, which is about one-seventh of pristine β-FeSi_2_. This is mainly due to strong phonon scattering from point defects and enhanced electron – phonon interactions at high carrier concentrations (10^21^–10^22^ cm^−3^). These combined scattering mechanisms significantly suppress the lattice thermal conductivity (*κ*_L_). In addition, Du et al. [47,61]. reported that doping with atoms having heavier atomic masses and larger atomic radius (e.g, Ru and Os) to the Fe site leads to mass and strain field fluctuation, resulting in the reduction in *κ*_L_. Possible band convergence effects may further contribute to the improved thermoelectric performance of metal-doped β-FeSi_2_.

## 5. Conclusions and prospects

In this review, we discussed structural transitions and strategies to enhance the thermoelectric performance of the metal-doped iron silicide (β-FeSi_2_) as an abundant and non-toxic compound. The formation of metallic secondary phases is highly sensitive to dopant type and concentration, and their presence often degrades thermoelectric transport. Electrical properties and power factor can be improved by doping elements with more valence electrons, while lattice thermal conductivity is effectively reduced through nanostructuring techniques (ball milling and stacking faults) and heavy element alloying (Ge, Ru, Os, and Ir). However, nanostructuring also introduces grain boundary scattering that lowers electrical conductivity, and excessive doping beyond solubility limits promotes metallic-phase formation, reducing the Seebeck coefficient.

Currently, the highest *ZT* value of 0.6 is achieved in 16% Ir-doped β-FeSi_2_, benefiting from its relatively high solubility limit, which enhances the power factor while lowering thermal conductivity. Further exploration of higher Ir concentrations, combined with optimized annealing conditions to stabilize the β-phase, may yield even higher *ZT* values. In addition, exploring higher concentrations of other heavy elements (Ru and Os) could possibly further enhance *ZT* due to the reduction in thermal conductivity. Other dopants, such as Co and Pt (n-type) and Mn and Al (p-type), are also promising to improve the power factor because of the enhancement of both electrical conductivity and Seebeck coefficient. Their higher solubility requires careful control of annealing temperature and duration to suppress secondary-phase formation.

Hence, three major strategies are crucial: (i) enhancing dopant solubility, (ii) incorporating heavier elements to disrupt phonon transport, and (iii) designing nanostructures with controlled grain sizes and stacking faults. Importantly, predicting and tuning phonon and electron mean free paths will be essential for engineering nanostructures that selectively scatter phonons while minimizing adverse effects on carrier mobility. Therefore, these approaches provide a pathway toward further improving the thermoelectric performance of β-FeSi_2_ and establishing it as a sustainable thermoelectric material.
